# Proteomic analysis of DEN and CCl_4_-induced hepatocellular carcinoma mouse model

**DOI:** 10.1038/s41598-024-58587-6

**Published:** 2024-04-05

**Authors:** Qian Zhang, Yuhui Liu, Liangliang Ren, Junqing Li, Weiran Lin, Lijuan Lou, Minghan Wang, Chaoying Li, Ying Jiang

**Affiliations:** 1 State Key Laboratory of Medicle Proteomics, Beijing Institute of Lifeomics, Beijing Proteome Research Center, National Center for Protein Sciences (Beijing), Beijing, 102206 China; 2 School of Basic Medical Science, Anhui Medical University, Hefei, 230032 China

**Keywords:** Hepatocellular carcinoma, Diethylnitrosamine, Carbon tetrachloride, Mouse model, Proteome, Cancer, Computational biology and bioinformatics, Molecular biology

## Abstract

Hepatocellular carcinoma (HCC) seriously threatens human health, mostly developed from liver fibrosis or cirrhosis. Since diethylnitrosamine (DEN) and carbon tetrachloride (CCl_4_)-induced HCC mouse model almost recapitulates the characteristic of HCC with fibrosis and inflammation, it is taken as an essential tool to investigate the pathogenesis of HCC. However, a comprehensive understanding of the protein expression profile of this model is little. In this study, we performed proteomic analysis of this model to elucidate its proteomic characteristics. Compared with normal liver tissues, 432 differentially expressed proteins (DEPs) were identified in tumor tissues, among which 365 were up-regulated and 67 were down-regulated. Through Gene Ontology (GO) analysis, Ingenuity Pathway Analysis (IPA), protein–protein interaction networks (PPI) analysis and Gene-set enrichment analysis (GSEA) analysis of DEPs, we identified two distinguishing features of DEN and CCl_4_-induced HCC mouse model in protein expression, the upregulation of actin cytoskeleton and branched-chain amino acids metabolic reprogramming. In addition, matching DEPs from the mouse model to homologous proteins in the human HCC cohort revealed that the DEN and CCl_4_-induced HCC mouse model was relatively similar to the subtype of HCC with poor prognosis. Finally, combining clinical information from the HCC cohort, we screened seven proteins with prognostic significance, SMAD2, PTPN1, PCNA, MTHFD1L, MBOAT7, FABP5, and AGRN. Overall, we provided proteomic data of the DEN and CCl_4_-induced HCC mouse model and highlighted the important proteins and pathways in it, contributing to the rational application of this model in HCC research.

## Introduction

With a high incidence and mortality rate, liver cancer is a global health challenge. According to the GLOBOCAN 2020 database (https://gco.iarc.fr/today/home), liver cancer is the third leading cause of cancer deaths (8.3% of total cancer deaths)^1^. HCC is the main form of liver cancer, comprising 75–85% of cases^1^. The occurrence of HCC is associated to a variety of factors, including HBV, HCV, aflatoxin, alcohol, diabetes, etc^2,3^. Mouse models are essential for the exploration of mechanisms and new therapeutic strategies for HCC^4^. A variety of animal models for HCC have been successfully generated to meet different experimental purposes^4^.

DEN and CCl_4_-induced HCC mouse model is a mostly common animal model with characteristics of fibrosis and inflammation, which is similar to human HCC^5^. DEN, a genotoxic carcinogen, is used to induce HCC in mice^6,7^, activated by cytochrome P450 in the liver to form alkylating agents leading to DNA damage and activating oxidative stress^7^. CCl_4_ is used extensively to induce the formation of fibrosis in multiple species^8,9^. The highly reactive free radical metabolites of CCl_4_ can cause liver damage by disrupting the integrity of cell membranes through lipid peroxidation, leading to compensatory cell proliferation and increased frequency of DNA mutations, which eventually develop into cancer^10–12^. The combination of DEN as an initiator and CCl_4_ as a promoting agent is usually reliable in inducing HCC^9,13–15^.

Since HCC is heterogeneous, the molecular characteristics of the selected animal models should be the most important consideration in preclinical studies^16,17^. Lee et al.^17^ found that DEN-induced liver cancer mouse model was more resemble to liver cancer populations with poor prognosis at the genome level. And Sun et al.^18^ investigated the expression characteristics of DEN and CCl_4_-induced HCC mouse model at the transcriptional level. Since proteins are functional executors and the genome and transcriptome cannot exactly mirror the protein landscape^19^, the proteomic profile of DEN and CCl_4_-induced HCC mouse model is urgent.

Here, DEN and CCl_4_-induced HCC mouse model was constructed, and the proteome of HCC and liver tissues was analyzed. This study provided valuable proteomic data for investigators and revealed the expression characteristics of DEN and CCl_4_-induced HCC mouse models at the protein level. In addition, combining the DEPs in this model with human HCC cohorts, SMAD2, PTPN1, PCNA, MTHFD1L, MBOAT7, FABP5, and AGRN were emphasized as potential prognostic proteins.

## Materials and methods

### Mouse model construction and tissue collection

C57BL/6J mice were purchased from Beijing Vital River Laboratory Animal Technology and then housed in animal facilities at the Phonix Center under a standard 12-h light/dark cycle with access to food and water ad libitum. C57BL/6J mice were injected intraperitoneally with DEN (N0258, Sigma) at a dose of 50 μg/g as the treatment group (T), while littermate mice were intraperitoneally injected with solvent as the control group. There were 4 male mice in each group. CCl_4_ was diluted 1: 4 in corn oil (ST1177, Beyotime Biotechnology). Two weeks later, the mice of the treatment group were treated with CCl_4_ (SINOPHARM, China) intraperitoneally at a dose of 0.5 μL/g, and the mice of the control group were treated with corn oil. Mice were sacrificed by cervical dislocation after 7 months of continuous induction of CCl_4_. Tumor tissues were harvested from the treatment group, and normal liver tissues were collected from the control group. Tissues for proteomic analysis were stored in a refrigerator at – 80 °C, and tissues for immunohistochemistry were fixed in 4% formaldehyde solution.

### Hematoxylin & eosin staining and immunohistochemistry

Tissues fixed in 4% paraformaldehyde were embedded in paraffin and sectioned. Liver sections (5 μm) were stained with hematoxylin and eosin (H&E) staining reagents after xylene dewaxing and gradient ethanol dehydration. And sections were subjected to antigen retrieval and incubated with primary antibody at room temperature for 1 h followed by overnight at 4 °C and incubated with anti-rabbit secondary antibody for 45 min at room temperature. Antibody include anti-Ki67 antibody (ab15580, abcam), anti-ACTA2 antibody (19245S, Cell Signaling Technology), anti-CD4 antibody (25229S, Cell Signaling Technology), anti-CD8 antibody (ab209775, abcam), anti-CD68 antibody (97778S, Cell Signaling Technology) and anti-F4/80 antibody (28463-1-AP, Proteintech). Images were captured using the Zeiss microscope (Wetzlar, Germany) and the percentage of positivity was analyzed with Fiji software.

### Protein extraction and quantitative proteomic analysis

Tissues, magnetic beads and tissue lysis solution (8 mol/L Urea in 0.1 mol/L trishydroxymethyl aminomethane, pH 8.5) containing protease inhibitors were mixed and ground using a High-Throughput Tissue Homogenizer (Scientz-48). Proteins were then extracted and concentrations were measured with Ultra-micro spectrophotometer (SimpliNano, Biochrom). Protein (30 μg) from each sample was separated by 10% SDS-PAGE and stained with Brilliant Blue G (B2025, Sigma) staining solution. Each gel lane was cut into small pieces and then decolored. Acetonitrile was added to dehydrate and remove the waste solution. Next, 10 mM DL-Dithiothreitol (0281-100G, VWR) solution was added and reacted for 1 h. After dehydration with acetonitrile, 50 mM iodoacetamide solution was added and reacted in the dark for 30 min. Similarly, acetonitrile was added to dehydrate and remove the waste solution. 0.01 μg/μL of trypsin (50 mM ammonium bicarbonate) was added and reacted at 37 °C for 12 h. The peptides were extracted with 0.5% trifluoroacetic acid and desalted with Stage Tips^20^.

Peptides were analyzed with liquid chromatography-mass spectrometry-tandem mass spectrometry (Q-Exactive HF LC–MS/MS) by the State Key Laboratory of Proteomics, Beijing Proteome Research Center (Beijing, China)^21,22^. The tandem mass spectra were analyzed with MaxQuant software (version 1.5.3.30) and searched against the human UniProt database (version 20140922, 20,193 sequences)^22–24^. And the false discovery rates (FDRs) of the peptide-spectrum matches (PSMs) peptides and proteins were 1%. The iBAQ intensity was used to represent protein expression for data analysis.

### Bioinformatic analysis

GO analysis (http://www.geneontology.org/)^25^, and GSEA of DEPs were performed using the R package “clusterProfiler”. The top enriched pathways were visualized using the R package “ggplot2”. The Benjamini–Hochberg procedure adjusted p-values (BH-adjusted *P*) < 0.05 was considered to have statistical significance.

DEPs were analyzed with Matascape (https://metascape.org/gp/index.html#/main/step1) to obtain PPI and molecular complex detections (MCODEs). PPI networks were imported into Cytoscape software, and the CytoHubba plugin was used to calculate the 10 proteins with the highest Degree values.

Gene names, protein expression averages, fold changes, and the BH-adjusted P value were uploaded to IPA, and species selected mice. Core analysis in IPA was performed to obtain Canonical Pathways, Upstream regulator (Data not shown) and Disease and Bio Functions (Data not shown).

### Western-blot

The total proteins (30 μg per sample) were subjected to 10% sodium dodecyl sulfate polyacrylamide gel electrophoresis and blotted onto nitrocellulose membranes. The membranes were blocked with 5% non-fat milk in Tris-buffered saline for 1 h at room temperature, then were incubated with rabbit anti-BCAT2 antibody (79764, Cell Signaling Technology), anti-BCKDHA antibody (90198, Cell Signaling Technology), anti-DBT antibody (ab151991, abcam) and anti-GAPDH (10494-1-AP, Proteintech) antibody overnight at 4℃, and secondary antibody for 1 h at room temperature. The blots were visualized with enhanced chemiluminescence Western Blotting Substrate. Finally, Image J software was used for protein quantification.

### Survival analysis

To explore the clinical value of DEPs, we converted these proteins to human homologs using the “biomaRt” R package, and performed prognostic analyses based on HCC cohorts previously studied in our laboratory^22^, contained proteomic data from 101 tumor tissues and 98 paired non-tumor tissues and clinical information from these patients. We matched protein expression of human homologous proteins corresponding to DEPs with prognostic information (Supplementary Table 10).

Prognostic analysis of the above proteins was identified with the “survival” and “survminer” R packages. Firstly, by Kaplan–Meier survival analysis, 42 proteins were identified to be associated with prognosis in Jiang et al.’s HCC cohort (5-year difference > 0.1, *P* < 0.05) (Supplementary Table 11). Next, the “survivalROC” R package was used to generate the receiver operating characteristic (ROC) curve and calculate the area under the ROC curve (AUC). In Jiang et al.’s HCC cohort, there were 30 proteins with favorable prognostic effects (5-year AUC value > 0.6, *P* < 0.05) (Supplementary Table 12).

### Statistical analysis

GraphPad Prism 9.0 was used to compare the data between the two groups by Student’s t-test. Data are presented as mean ± standard deviation. P < 0.05 was considered statistically significant.

## Results

### Proteome analysis of DEN and CCl_4_-induced HCC mouse model

**Figure 1:**
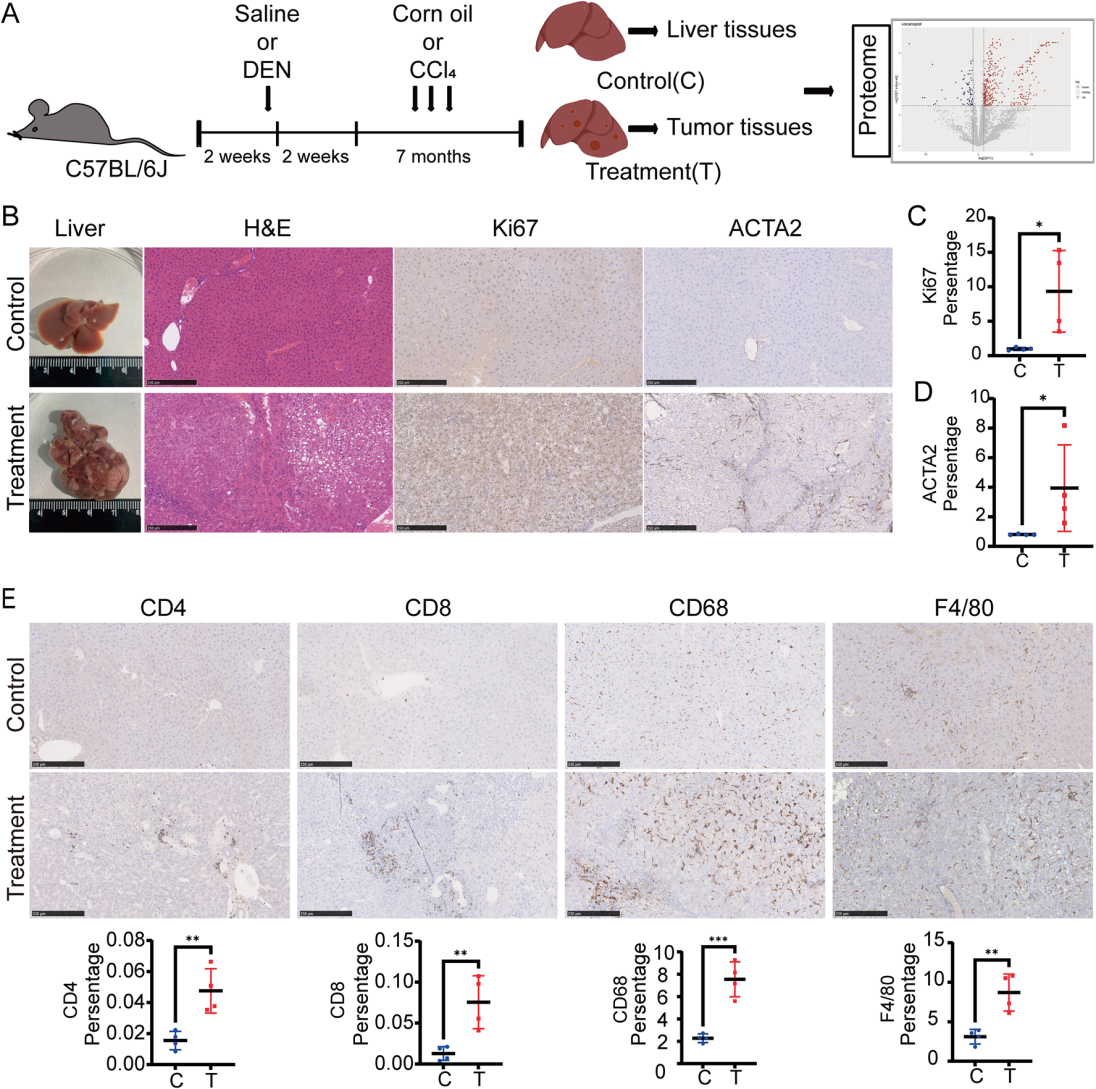
Construction of DEN and CCl_4_-induced HCC mouse model. (**A**) Schematic diagram of the construction and proteomic analysis of DEN and CCl_4_-induced HCC models in C57BL/6J mice. (**B**) Representative images of mouse liver, H&E staining and immunohistochemical staining of Ki67 and ACTA2 (C = control, T = treatment, n = 4). (**C**) Quantification of the percentage of positive area for Ki67 immunohistochemical staining (n = 4). (**D**) Quantification of the percentage of positive cells stained for ACTA2 immunohistochemical staining (n = 4). (**E**) Representative images of immunohistochemical CD4, CD8, CD68, F4/80 of liver tissues and tumor tissues. (C = control, T = treatment, n = 4) scale bar: 250 μm, *P < 0.05, **P < 0.01 and ***P < 0.001.

Following the classical method^5,26^, we constructed DEN and CCl_4_-induced HCC mouse model. Normal liver tissues from the control mice and tumor tissues from the treated mice were collected for proteomic analysis (Fig. 1A). In DEN and CCl_4_-induced mouse model, multiple tumors developed in the liver (Fig. 1B). H&E staining showed hepatocytes in liver tissues of control mice were neatly arranged with normal cell morphology, but tumor tissues of DEN and CCl_4_-induced mice were disordered with disorganized morphology, large nuclei, and obvious immune infiltration, suggesting that DEN and CCl_4_ induced carcinogenesis in the liver of mice (Fig. 1B). Immunohistochemical analysis showed that the percentage of positive area for the cell proliferation marker Ki67 and the percentage of positive cells for the typical liver fibrosis marker ACTA2^27^ were significantly increased in tumor tissue compared with normal liver tissues (Fig. 1B–D). Immunohistochemical results showed a significant increase in the infiltration of CD4+ T cells, CD8+ T cells, CD68+ macrophages, and F4/80+ Kuffer cells in tumor tissues (Fig. 1E), which suggested a high degree of immune infiltration in the tumor tissues of DEN and CCl_4_-induced HCC mouse models. These results demonstrated that DEN and CCl_4_-induced HCC mouse model was successfully constructed, and the tumor tissues exhibited cell proliferation and fibrosis characteristics.

**Figure 2:**
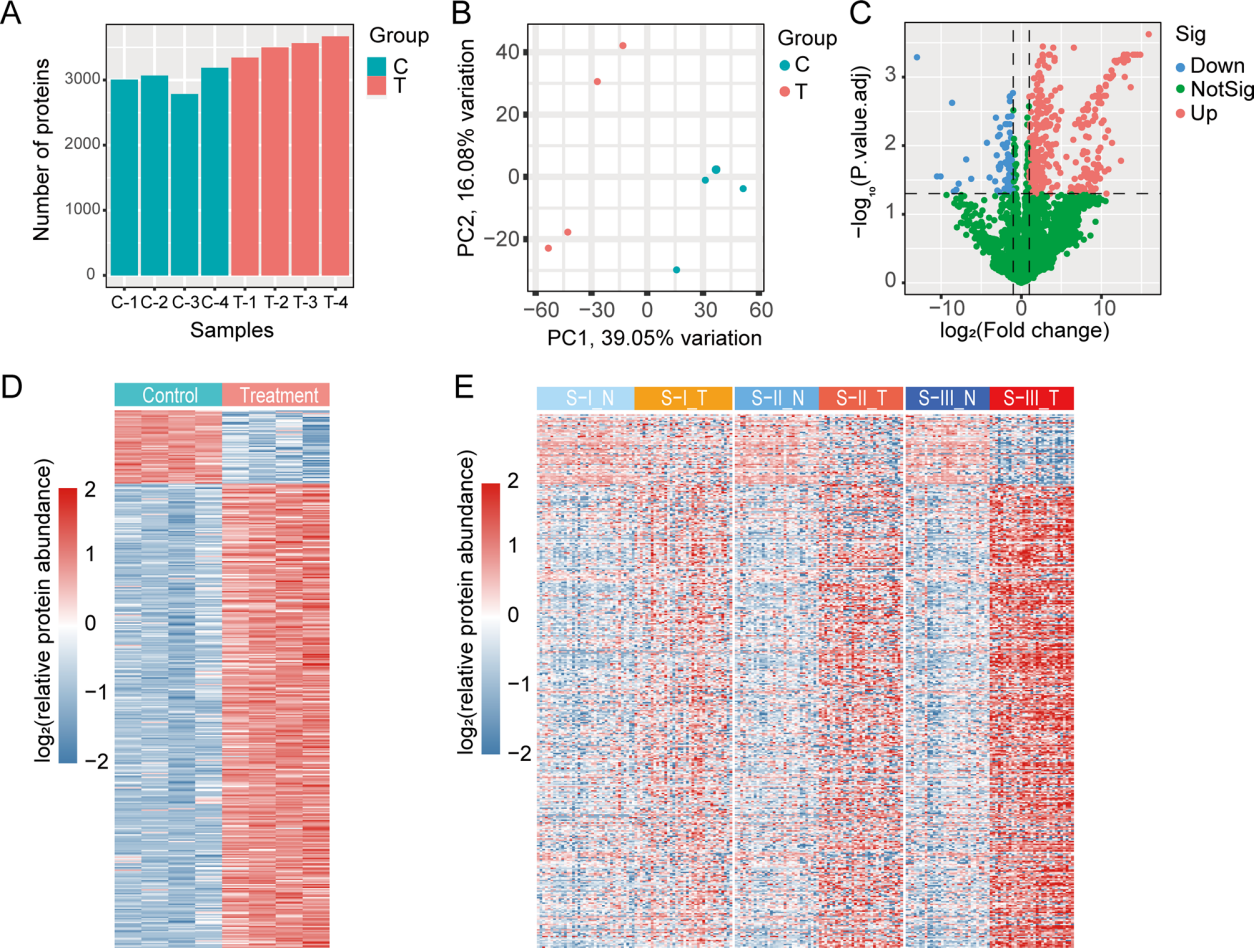
Proteomic analysis of DEN and CCl_4_-induced HCC mouse model. (**A**) The number of proteins identified in each sample. (**B**) PCA clearly separates liver tissue samples from control mice (C, green) and tumor tissue samples from model mice (T, red). (**C**) Volcano plot of DEPs. Blue dots represent down-regulated proteins, red dots represent up-regulated proteins, and green dots represent proteins with insignificant differences (|log2 (fold change) |≥ 1, *P*.adj < 0.05). *P < 0.05, **P < 0.01 and ***P < 0.001. (**D**) Heatmap of DEPs expression in liver tissue samples from control mice (C, green) and tumor tissue samples from model mice (T, red). (**E**) Heatmap of DEPs homologous protein expression in non-tumor (N, green) and tumor tissues (T, red) of three proteomic subtypes of Jiang et al.’s HCC cohort, S-I (n = 36), S-II (n = 31) and S-III (n = 31). R software (version 4.2.1) was used to create heatmaps.

To characterize the protein expression in DEN and CCl_4_-induced HCC mouse model, we performed total protein extraction and quantitative proteomic analysis on tumor tissue from 4 model mice and liver tissue from 4 control mice, respectively. We totally identified and quantified 4383 proteins, of which 3960 were expressed in at least 2 of a group of 4 mice (Supplementary Table 1, Fig. 2A). Principal component analysis (PCA) revealed significant spatial separation between tumor tissue and liver tissue (Fig. 2B). Filtered by |log_2_ (fold change) |≥ 1 and the BH-adjusted *P* value < 0.05, a total of 432 differentially expressed proteins (DEPs) were obtained, of which 365 proteins were significantly up-regulated and 67 proteins were significantly down-regulated (Fig. 2C, D).

Jiang et al.^22^ utilized proteome to analyze paired tumor and non-tumor tissues from early-stage HCC patients. Their findings categorized the cohort into three subtypes: S-I with hepatocyte-like traits, S-II with proliferative traits, and S-III showcasing proliferation, aggressiveness, and heightened immune infiltration, in which S-III with the worst prognosis. Based on the data from the above study, we performed a differential expression protein analysis (|log2 (fold change) |≥ 1, *P*.adj < 0.05) of tumor and non-tumor tissues of the three subtypes of HCC (Supplementary Tables 2–4). And the differentially expressed proteins of S-III overlapped more with those of DEN and CCl4-induced HCC mouse model (Supplementary Fig. 1A). To determine the value of DEN and CCl_4_-induced HCC mouse model's DEPs for clinical studies, we used heatmaps to demonstrate the expression of human homologs of these proteins in tumor and non-tumor tissues of the three subtypes of HCC (Fig. 2E). In addition, Pearson correlation analysis showed that expression of these proteins in this mouse model correlated more strongly with expression in S-III compared to S-I and S-II (Supplementary Fig. 1B). These results suggested that the DEN and CCl_4_-induced HCC mouse model may be more suitable for the study of S-III subtype HCC.

### Upregulation of actin cytoskeleton in DEN and CCl_4_-induced HCC mouse model

**Figure 3:**
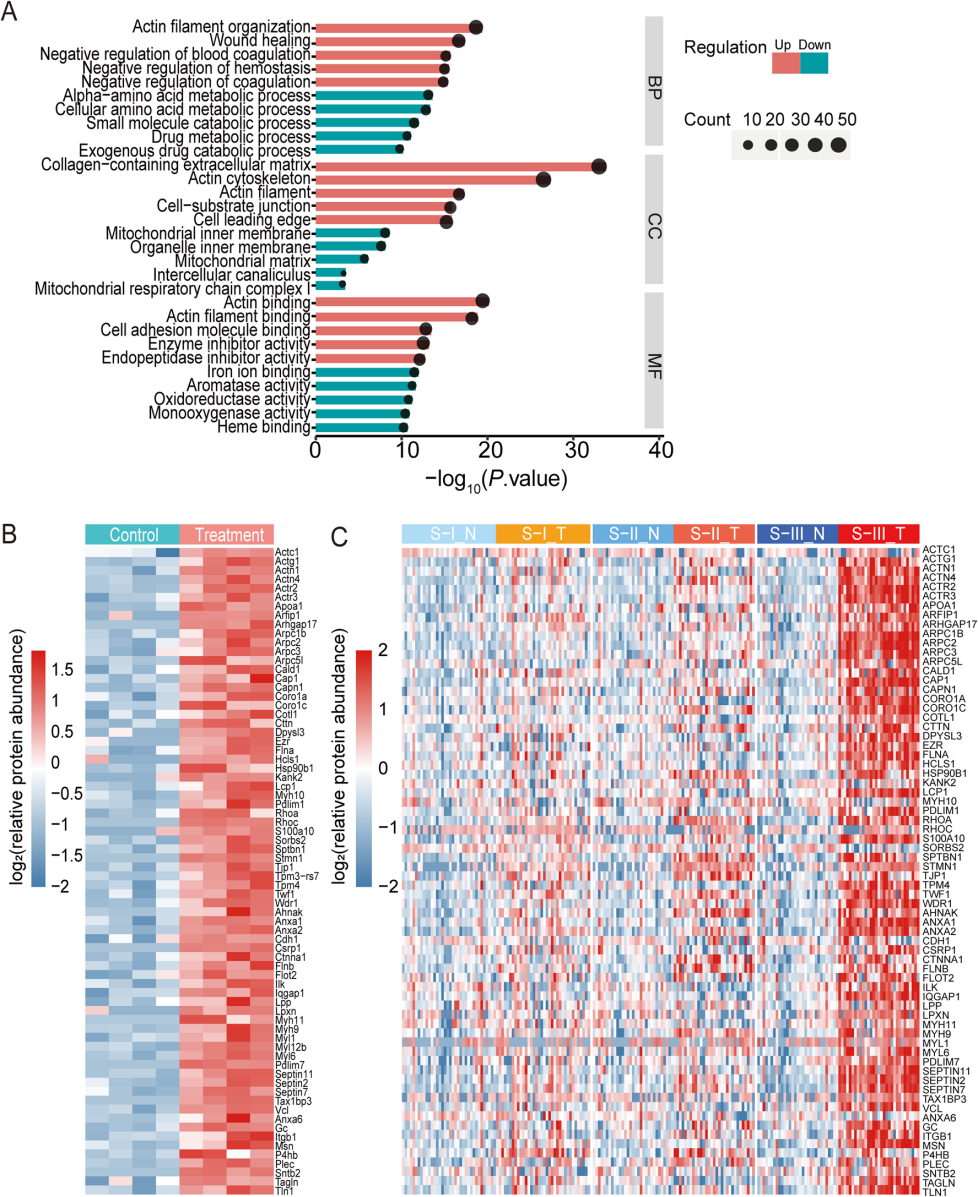
Upregulation of actin cytoskeleton in DEN and CCl_4_-induced HCC mouse model. (**A**) The top significant terms of biological processes from GO analysis. Blue bars represent downward entries and red bars represent upward entries, *P*. adj < 0.05. (**B**) Heatmap of actin cytoskeleton-associated proteins expression in liver tissue samples from control mice (C, green) and tumor tissue samples from model mice (T, red). (**C**) Heatmap of actin cytoskeleton-associated proteins homologous protein expression in non-tumor (N, green) and tumor tissues (T, red) of three proteomic subtypes of Jiang et al.’s HCC cohort, S-I (n = 36), S-II (n = 31) and S-III (n = 31). R software (version 4.2.1) was used to create heatmaps.

To reveal the biological characteristics of tumor tissues in DEN and CCl_4_-induced mouse model, we performed functional enrichment analysis of DEPs. First, GO analysis was applied in terms of biological processes (BP), molecular function (MF), and cellular composition (CC) (Fig. 3A, Supplementary Table 5). The up-regulated proteins were mainly involved in the actin filament organization process, collagen-containing extracellular matrix, actin cytoskeleton, and actin binding functions. The down-regulated proteins were largely concentrated in alpha-amino acid metabolic process, the ligase activity, and the mitochondrial inner membrane. IPA analysis suggested that the actin cytoskeletal signaling pathway is significantly activated (Supplementary Figs. 2, 3 and Supplementary Table 6). In addition, pathways that positively regulate actin cytoskeleton were activated, such as Signaling by Rho Family GTPases^28,29^, Integrin Signaling^30^, and ILK Signaling^31^. However, the pathway RHOGDI Signaling, which negatively regulates actin cytoskeleton^32^, was inhibited.

Therefore, we focused on the expression of actin cytoskeletal related proteins in HCC. We used heatmaps to demonstrate the expression of proteins in the actin cytoskeleton, actin filament organization processes, and actin-binding function entries in DEN and CCl_4_-induced HCC mouse models and the subtypes S-I, S-II and S-III (Fig. 3B, C). The results showed that actin cytoskeletal related proteins, which were significantly upregulated in DEN and CCl_4_-induced HCC mouse models, were similarly significantly upregulated in S-III tumor tissues. However, it was not significant in S-I or S-II.

Subsequently, to investigate important protein interactions in the DEN and CCl_4_-induced HCC mouse model, we analyzed DEPs with Metascape to generate PPI networks (Supplementary Fig. 4A)^33^. The core proteins of the PPI network were analyzed by Cytoscape, the top 10 proteins in terms of Degree value (Supplementary Fig. 4B, Supplementary Table 7), Integrin beta-1 (Itgb1), Actin cytoplasmic 2 (Actg1), Integrin beta-2 (Itgb2), Transforming protein RhoA (Rhoa), Actin alpha cardiac muscle 1 (Actc1), Rho-related GTP-binding protein RhoC (Rhoc), Vinculin (Vcl), Integrin alpha-6 (Itga6), Actin-related protein 3 (Actr3), and Actin-related protein 2 (Actr2), which were involved in the formation and regulation of the actin cytoskeleton. Next, this PPI network was calculated by Metascape's MCODE algorithm and identified 23 MCODEs (Supplementary Fig. 4C, Supplementary Table 8), which were densely connected neighborhoods of the proteins^34^. We focused on top 3 MCODEs with scores greater than 4, and performed GO enrichment analysis on the proteins among them, and the top 3 terms were shown in Table1. The proteins of MCODE1 were participated in actin cytoskeleton organization, actin filament organization process, and supramolecular fiber organization.

**Table 1:** Top 3 best *P*-value terms for GO enrichment analysis of the 3 MCODE networks (MCODE Score > 4).

MCODE	Score	GO	Description	Log (*P*)
MCODE_1	9.2	GO:0030036	Actin cytoskeleton organization	− 19.7
		GO:0030029	Actin filament-based process	− 19.1
		GO:0097435	Supramolecular fiber organization	− 15.7
MCODE_3	4.69	mmu05205	Proteoglycans in cancer-Mus musculus (house mouse)	− 12
		GO:1903829	Positive regulation of protein localization	− 7.4
		GO:0032386	Regulation of intracellular transport	− 6.9
MCODE_6	4	GO:0034369	Plasma lipoprotein particle remodeling	− 19.3
		GO:0,034,368	Protein-lipid complex remodeling	− 19.3
		GO:0034367	Protein-containing complex remodeling	− 19

Taken together, through GO analysis, IPA analysis and PPI analysis, we observed a significant upregulation of actin cytoskeleton, which may play an important role in HCC development.

### BCAAs metabolic reprogramming in DEN and CCl_4_-induced HCC mouse model

**Figure 4:**
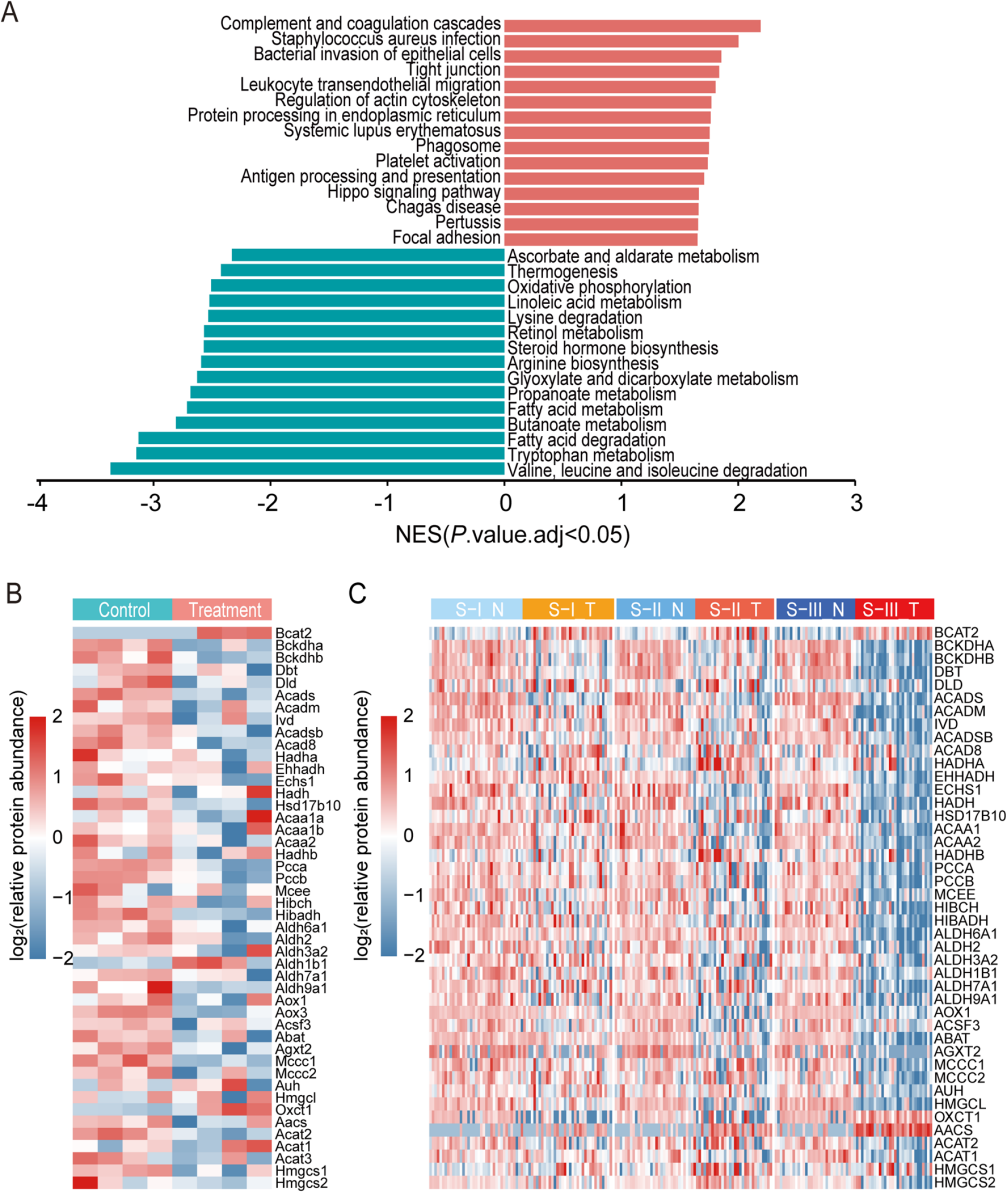
BCAAs metabolic reprogramming in DEN and CCl_4_-induced HCC mouse model. (**A**) Top pathways from GSEA (KEGG pathway analysis) of DEPs. Blue bars represent downward pathways and red bars represent upward pathways, *P*.adj < 0.05. (**B**) Heatmap of valine, leucine and isoleucine degradation pathway expression in liver tissue samples from control mice (C, green) and tumor tissue samples from model mice (T, red). (**C**) Heatmap of valine, leucine and isoleucine degradation pathway expression in non-tumor (N, green) and tumor tissues (T, red) of three proteomic subtypes of Jiang et al.’s HCC cohort, S-I (n = 36), S-II (n = 31) and S-III (n = 31). R software (version 4.2.1) was used to create heatmaps.

**Figure 5:**
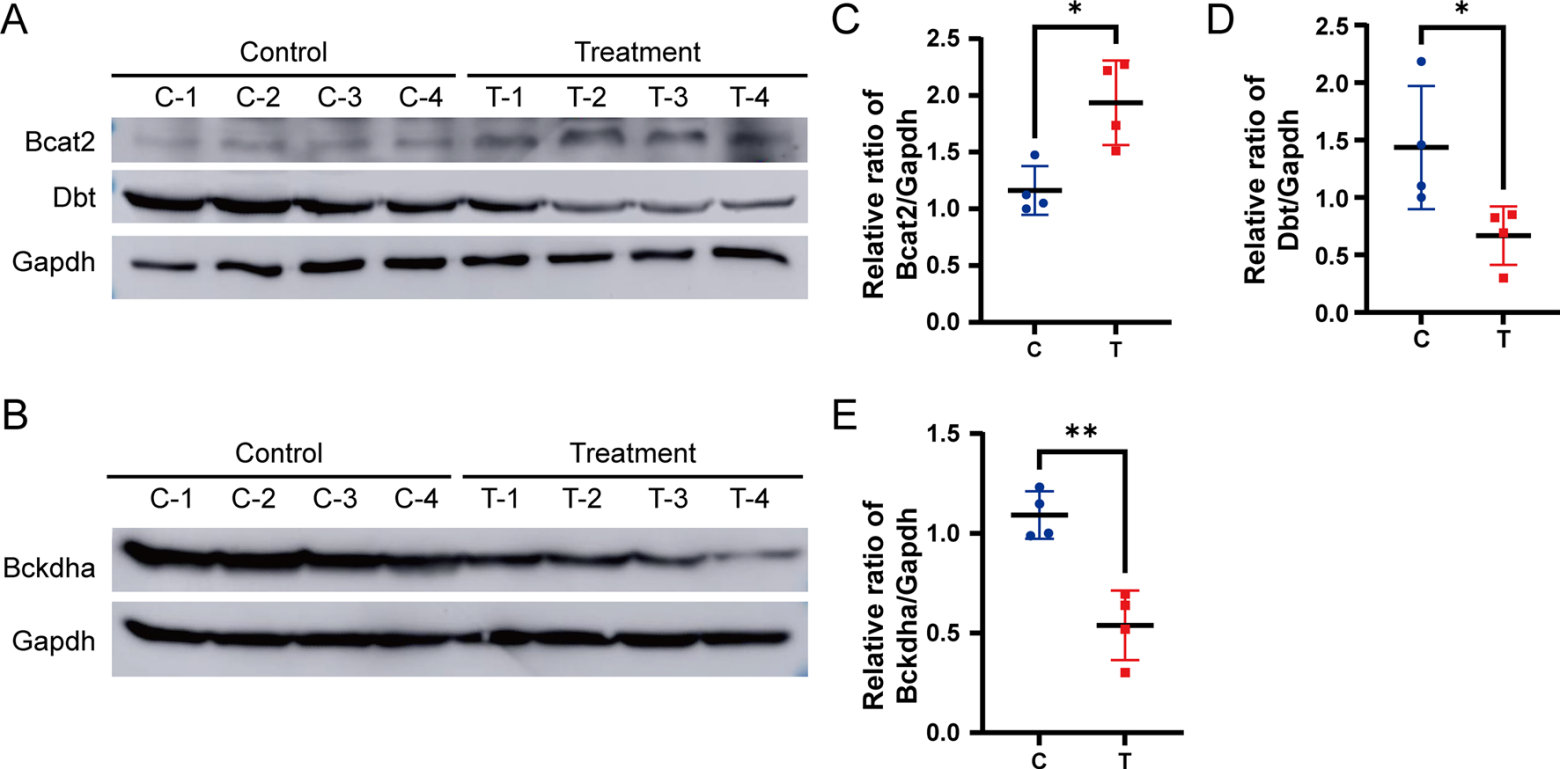
Immunoblots of selected BCAA metabolic enzymes. (**A**, **B**) Immunoblots of Bcat2, Dbt (**A**) and Bckdha (**B**) in liver tissue samples from control mice and tumor tissue samples from model mice. Images of full-length immunoblots are shown in Supplementary Fig. 7. (**C**–**E**) Quantification ratios of Bcat2 (**C**), Dbt (**D**) and Bckdha (**E**) in the above tissues with Gapdh as a loading control (C = control, T = treatment, n = 4). *P < 0.05, **P < 0.01 and ***P < 0.001.

Furthermore, GSEA analysis (Kyoto Encyclopedia of Genes and Genomes, KEGG^35^, http://www.genome.jp/kegg/) showed that the pathway of valine, leucine, and isoleucine (Branched-chain amino acids, BCAAs) degradation was significantly down-regulated (Fig. 4A). BCAAs degradation is composed of two steps: (1) Branched-chain amino acid transaminases (BCAT1 and BCAT2) deaminate BCAAs to branched-chain α-keto acids (BCKAs), and the reaction is reversible. (2) BCKAs are oxidized by branched chain ketoacid dehydrogenase (BCKDH) complex and its downstream catabolic enzymes, and finally enter the tricarboxylic acid cycle^36^. Our study shows that the expression of Branched-chain amino acid transaminase 2 (BCAT2) was upregulated and oxidative catabolic enzymes of BCKAs are downregulated in DEN and CCl_4_-induced HCC mouse models (Fig. 4B). Attractively, BCAAs metabolic reprogramming was more clearly observed in S-III tumor tissues than in S-I, S-II tumor tissues (Fig. 4C). Finally, this phenomenon was also confirmed by western-blot experiments (Fig. 5). Compared with normal mouse liver tissues, the expression of BCAT2 was higher, while the expressions of BCKDHA and DBT were lower in the tumor tissues of DEN and CCl_4_-induced HCC mouse models.

### Prognostic value of DEPs for HCC in DEN and CCl_4_-induced HCC mouse model

To identify the prognostic proteins, we combined the DEPs of the DEN and CCl_4_-induced HCC mouse model with proteomic data from Jiang et al.’s study^22^ (Supplementary Fig. 5). Based on the HCC clinical sample information, potential prognostic markers were screened by the conditions of significant correlation with survival (P < 0.05), difference in 5-year survival between high and low expression groups greater than 0.1, and AUC value of 5-year ROC curve greater than 0.6. Jiang et al.’s study contained proteomic data from 100 paired tumor tissues and non-tumor tissues and clinical information from these patients. We matched protein expression of human homologous proteins corresponding to DEPs with prognostic information (Supplementary Table 10).

Firstly, by Kaplan–Meier survival analysis, 42 proteins were identified to be associated with prognosis in Jiang et al.’s cohort (5-year difference > 0.1, *P* < 0.05) (Supplementary Table 11). Next, there were 30 proteins with favorable prognostic effects (5-year AUC value > 0.6, *P* < 0.05) (Supplementary Table 12). Finally, seven proteins from the above 30 proteins were screened for differential expression in non-tumor and tumor tissues of HCC clinical samples by |log2 (fold change) |≥ 1 and the BH-adjusted *P* value < 0.05, Mothers against decapentaplegic homolog 2 (SMAD2), tyrosine-protein phosphatase non-receptor type 1 (PTPN1), Proliferating cell nuclear antigen (PCNA), monofunctional C1-tetrahydrofolate synthase, mitochondrial (MTHFD1L), Lysophospholipid acyltransferase 7(MBOAT7), Fatty acid-binding protein 5 (FABP5) and Agrin (AGRN) (Supplementary Fig. 6).

**Figure 6:**
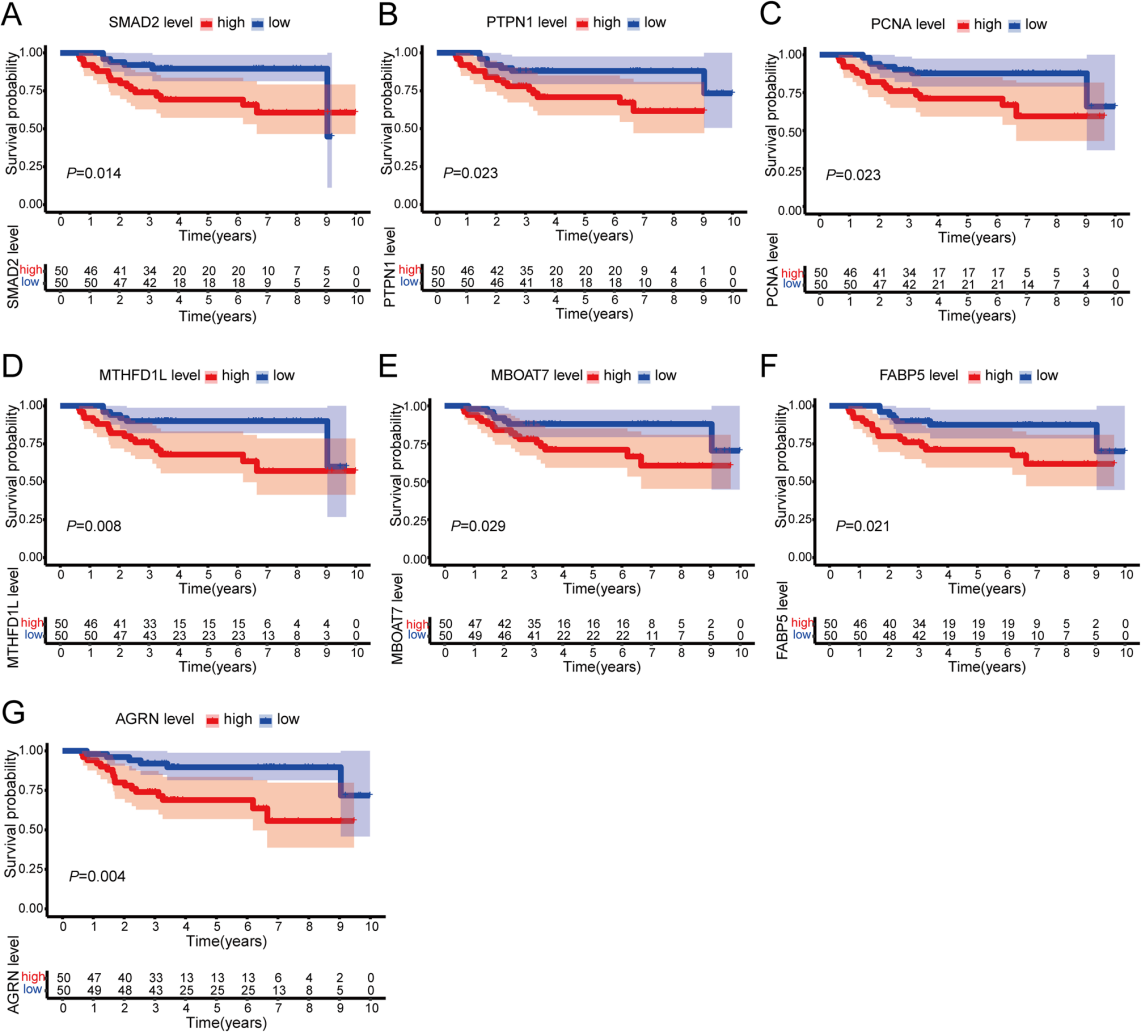
Overall survival curves for seven proteins with significant prognostic value. (**A**–**G**) Overall survival curves of (**A**) MAD2, (**B**) PTPN1, (**C**) PCNA, (**D**) MTHFD1L, (**E**) MBOAT7, (**F**) FABP5, (**G**) AGRN in Jiang et al.’s HCC cohort.

**Figure 7:**
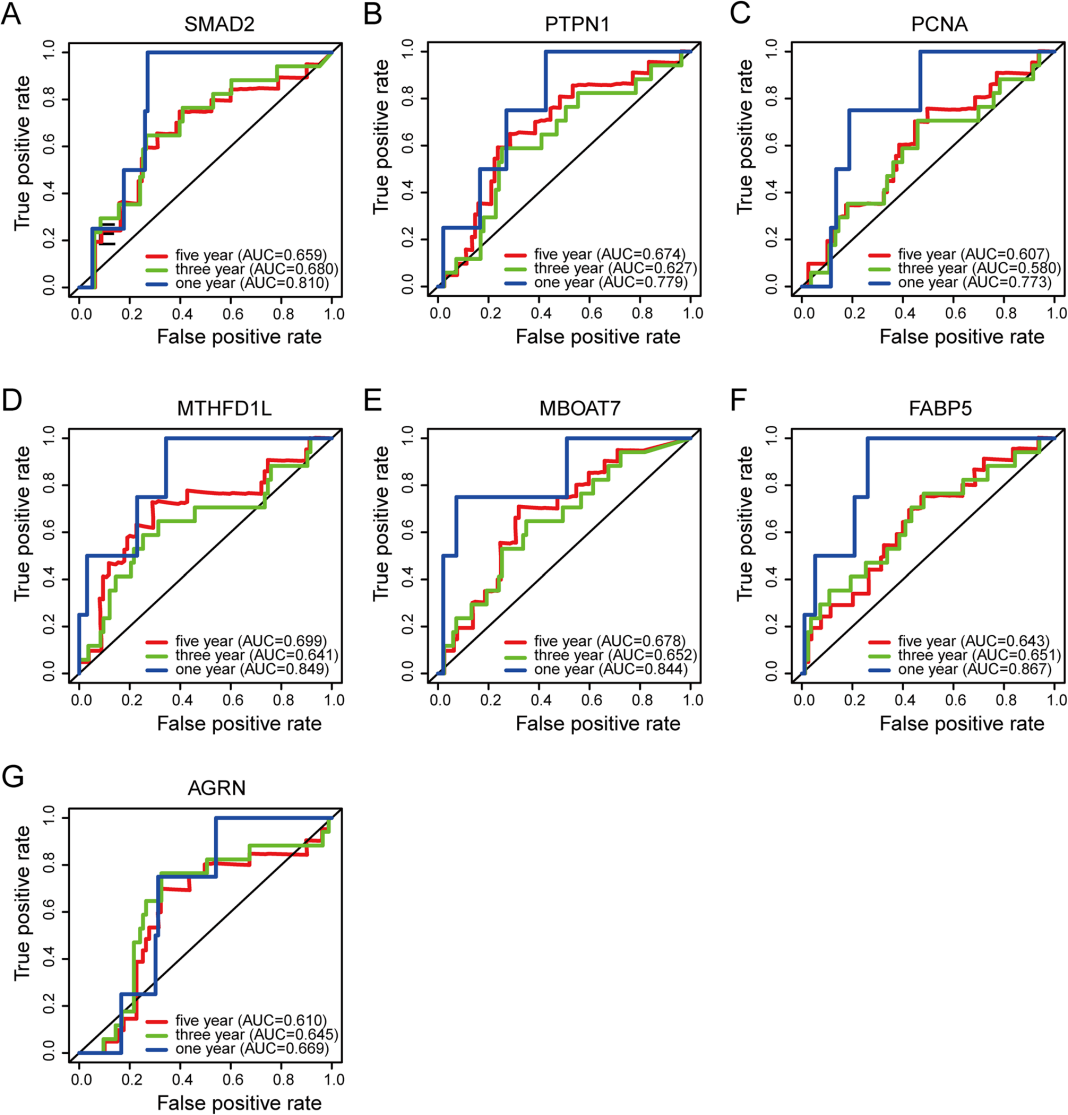
ROC curves for seven proteins with significant prognostic value. (**A**–**G**) ROC curves of (**A**) MAD2, (**B**) PTPN1, (**C**) PCNA, (**D**) MTHFD1L, (**E**) MBOAT7, (**F**) FABP5, (**G**) AGRN in Jiang et al.’s HCC cohort.

These seven proteins were significantly highly expressed in tumor tissues of DEN and CCl_4_-induced HCC mice (Supplementary Fig. 6A–G). Consistently, these seven proteins were highly expressed in tumor tissues of HCC patients compared to non-tumor tissues (Supplementary Fig. 6H–N). Overall survival curves (Fig. 6) and ROC curves (Fig. 7) for seven proteins showed that high expression of SMAD2, PTPN1, PCNA, MTHFD1L, MBOAT7, FABP5, and AGRN was associated with poor prognosis of HCC.

## Discussion

HCC is heterogeneous due to the complexity of etiologies and the multistep nature of the disease process. Therefore, one animal model of HCC cannot be suitable for all HCC studies. However, as an important tool for HCC research, which type of HCC is DEN and CCl_4_-induced HCC mouse model suitable for study? One of the important tasks of this study is to provide HCC investigators with proteomic data of the DEN and CCl_4_-induced HCC mouse model as a basis for their HCC mouse model selection.

Cross-species comparison of gene expression patterns in HCC reveals that DEN-induced hepatocellular carcinoma in mice is most similar to that of human HCC in the poorer survival group^17^. Consistently, our results showed that the expression of DEPs between DEN and CCl_4_-induced HCC mouse model tumor tissues and normal liver tissues was relatively similar to that of S-III, which is a poor prognostic subtype. Moreover, the two distinct features highlighted in DEN and CCl_4_-induced HCC mouse model tumor tissues, up-regulation actin cytoskeleton and BCAA metabolic reprogramming, were similarly expressed in S-III. These evidences above suggest that DEN and CCl_4_-induced HCC mouse model is more suitable for the study of S-III subtype HCC.

In imperfections, the mouse model proteomics data cannot comprehensively mimic S-III subtype HCC due to the limitations of multiple factors, such as cross-species, complexity of the disease process, and experimental techniques. For example, BCAAs metabolic reprogramming was exhibited in tumor tissues of both S-III and DEN and CCl_4_-induced HCC mouse models, but upregulation of BCAT1 and BCAT2 was detected in tumor tissues of S-III, whereas only upregulation of BCAT2 was detected in DEN and CCl_4_-induced HCC mouse model. The possible reason for this is the extremely low expression of BCAT1 in tumor tissues of DEN and CCl_4_-induced HCC mouse models, suggesting that DEN and CCl_4_-induced HCC mouse models are more suitable for the study of BCAT2 rather than BCAT1 in HCC. By analogy, based on the proteomic data we provide, researchers can get a clear picture of the expression of the proteins and related pathways under study as a reference for determining whether the model is a preferred choice.

Sun et al.^18^ performed a comprehensive transcriptomic analysis of DEN and CCl_4_-induced HCC mouse models by RNA-seq and identified 2033 up-regulated and 841 down-regulated genes. They then performed a functional enrichment analysis showing that these differential genes were enriched in the KEGG pathways metabolic pathway for chemical carcinogenesis, steroid hormone biosynthesis, retinol metabolism, and cytochrome P450. Similarly, in the proteomic data of our study, upregulation of the pathway of Chemical carcinogenesis-DNA adducts and metabolic disorders were observed (Supplementary Table 9). However, proteomic data analysis revealed significant alterations in the actin cytoskeleton of the tumor tissue, a feature not presented in the transcriptomic data analysis of the Sun et al. study^18^. Admittedly, alterations in transcript levels are not equivalent to differences in protein levels, which directly affect the phenotype. Therefore, we provide proteomic data as a new addition to the molecular characterization of DEN and CCl_4_-induced HCC mouse models.

Our data showed significant upregulation of the actin cytoskeleton in DEN and CCl_4_-induced HCC mouse models tumor tissues. Previous studies have proposed that DEN and CCl_4_ cause liver injury by activating hepatic stellate cells (HSCs) leading to the genesis of a robust actin cytoskeleton^37,38^, and the actin cytoskeleton functions in signal transduction to promote increased expression of extracellular matrix^38–40^. Consistent with this, we observed significant activation of HSCs and the upregulation of collagen-containing extracellular matrix in tumor tissues of this model (Figs. 1B, 3A). The actin cytoskeleton is involved in many cellular processes and maintains the stability of the intracellular environment^41,42^. Actin and actin-binding proteins are involved in all processes of cancer including carcinogenesis, migration, invasion and vascularization^43^. Given the magnitude contribution of the cytoskeleton to HCC, targeting components of the cytoskeleton as a therapeutic strategy is an attractive concept^44,45^. Alterations in the actin cytoskeleton may play an important role in HCC and are worthy of further investigation. And DEN and CCl_4_-induced HCC mouse model is suitable for studying actin cytoskeleton remodeling in HCC.

The liver is an important organ in the systemic metabolism of BCAAs, with relatively high expression of BCKDH complex and relatively low expression of BCATs^36,46^. Intriguingly, our proteomic data showed that in contrast to normal liver, DEN and CCl_4_-induced HCC mice tumor tissues expressing BCKDH complex were down-regulated while expressing BCAT2 was up-regulated. In Russell E. Ericksen’s study^47^, consistent results were observed by transcriptome, metabolomics and Western blot that catabolism of BCKAs was inhibited in HCC tumor tissues. They proposed that loss of BCAAs catabolism leads to BCAAs accumulation, enhances mTORC1 activity and promotes tumorigenesis and progression. On the contrary, Yang et al.^48^ found that glutamine deprivation in HCC tumor tissues leads to high expression and dephosphorylation of BCKDHA and promotes catabolism of BCAAs. In either case, the metabolic vulnerability caused by BCAAs metabolic reprogramming could be a potential target for HCC therapy. Obviously, the DEN and CCl_4_-induced HCC mouse model is suitable for the study of HCC in which the metabolism of BCAAs is inhibited. However, studies exploring the role and mechanism of BCAT2 in HCC are lacking. Chen Ding’s study^49^ found that in normal mouse liver, BCAT2 was mainly expressed in non-parenchymal cells, while the enzyme for oxidative catabolism of BCKAs was mainly expressed in parenchymal cells. We therefore speculate that the upregulation of BCAT2 may be partly attributable to the increase in non-parenchymal cells such as HSCs and macrophages, which requires experimental demonstration.

Combined with the human HCC cohorts, SMAD2, PTPN1, PCNA, MTHFD1L, MBOAT7, FABP5, and AGRN were screened as potential prognostic markers. SMAD2, one of the receptor-activated SMADs, binds to the shared partner SMAD4 after TGFβR1-mediated phosphorylation and followed by translocation into the nucleus, where these complexes activate transcription of specific genes^50^. During chronic inflammatory stimulation, SMAD2 is involved in fibro-carcinogenesis of the TGFβ signaling pathway^51^.*PTPN1* gene encodes protein tyrosine phosphatase 1B (PTP1B), which is a classical non-transmembrane protein tyrosine phosphatase, and its role in cancer progression is controversial^52^. FanG Yuan et al.’s study had shown that targeted inhibition of PTP1B could reduce the ability of proliferation, migration, and invasion of HCC cell lines^53^. PCNA is expressed in the nuclei of normal proliferating cells and tumor cells, involved in DNA replication, and is an important protein for cell proliferation^54–56^. Ma et al.^57^ showed that PCNA expression is increased in HCC tissues and that high PCNA expression can be used as an independent predictor of disease-free survival and overall survival. MTHFD1L is an important enzyme of the folate cycle^58^. Lee et al.^59^ reported that MTHFD1L was significantly highly expressed in tumor tissues and patients with high MTHFD1L expression had poor clinical outcomes in HCC. Several studies have shown that genetic polymorphisms in MTHFD1L are associated with the development of HCC^60–64^. However few studies have reported the effect of MTHFD1L on HCC at the protein level. Previous studies^64–67^ reported that FABP5 expression was associated with poor prognosis, which is consistent with our results. FABP5 promotes HCC development through multiple pathways, including binding to Hif1α to drive reprogramming of lipid metabolism^65^, induction of epithelial-mesenchymal transition^64^, tumor immune escape^66,68^, and angiogenesis^69^. AGRN has also been reported as a prognostic marker for HCC^70^ and promotes the proliferation and migration of hepatocellular carcinoma cells^71^. Overall, the seven HCC prognostic markers we screened are valuable for further research.

In summary, we performed proteomic analysis of DEN and CCl_4_-induced HCC mouse models to clarify the proteomic characteristics of this model at the protein level, which would facilitate the application of the model to provide possible insights into the molecular mechanism of HCC. In addition, we analyzed the proteomics of this mouse model in conjunction with a human HCC cohort and propose that the protein expression of this model is relatively similar to that of S-III and found seven proteins significantly associated with poor prognosis.

### Ethics approval and consent to participate

All animal protocols were in accordance with ARRIVE guidelines and were approved by the Committee on Animal Research and Ethics of Phonix Center, China (Institutional Animal Care and Use Committee ID-20220705-41MT). The methods of this study were informed to all the authors and were conducted in accordance with relevant guidelines and regulations.

### Supplementary Information

Supplementary Information.

## Data Availability

The clinical information and proteomic data of human HCC used in this study were obtained from the article published by Ying Jiang et al^22^. Proteomic data for mouse HCC are available in the paper and its supplemental data. Proteomic data for mouse HCC are available in the paper and its supplemental data. And the raw and result files have been uploaded to the iProX database for our proteome data sets (www.iprox.org, accession number, PXD049148)72,73.
