# Supplementary material for: Proteomic analysis of DEN and CCl4-induced hepatocellular carcinoma mouse model
Source: Sci Rep. 2024 Apr 5;14:8013. doi: 10.1038/s41598-024-58587-6 (PMC10997670; doi:10.1038/s41598-024-58587-6)
Supplement: Supplementary file 1 — Supplementary Information. [file 41598_2024_58587_MOESM1_ESM.zip › Supplementary Figures.docx]

**Proteomic analysis of DEN and CCl_4_-induced hepatocellular carcinoma** **mouse model**

**Qian Zhang^a^, Yuhui Liu^a^, Liangliang Ren^a^, Junqing Li^a,b^, Weiran Lin^a^, Lijuan Lou^a^, Minghan Wang^a^, Chaoying Li^a^, Ying Jiang^a,b*^**

**^a^State Key Laboratory of Proteomics, Beijing Proteome Research Center, National Center for Protein Sciences (Beijing), Beijing Institute of Lifeomics, Beijing, 102206, China**

**^b^School of Basic Medical Science, Anhui Medical University, Hefei 230032, China**

**^*^Corresponding author. State Key Laboratory of Proteomics, Beijing Proteome Research Center, National Center for Protein Sciences (Beijing), Beijing Institute of Lifeomics, Beijing, 102206, China**

**E-mail address:** [**jiangying304@hotmail.com**](mailto:jiangying304@hotmail.com)


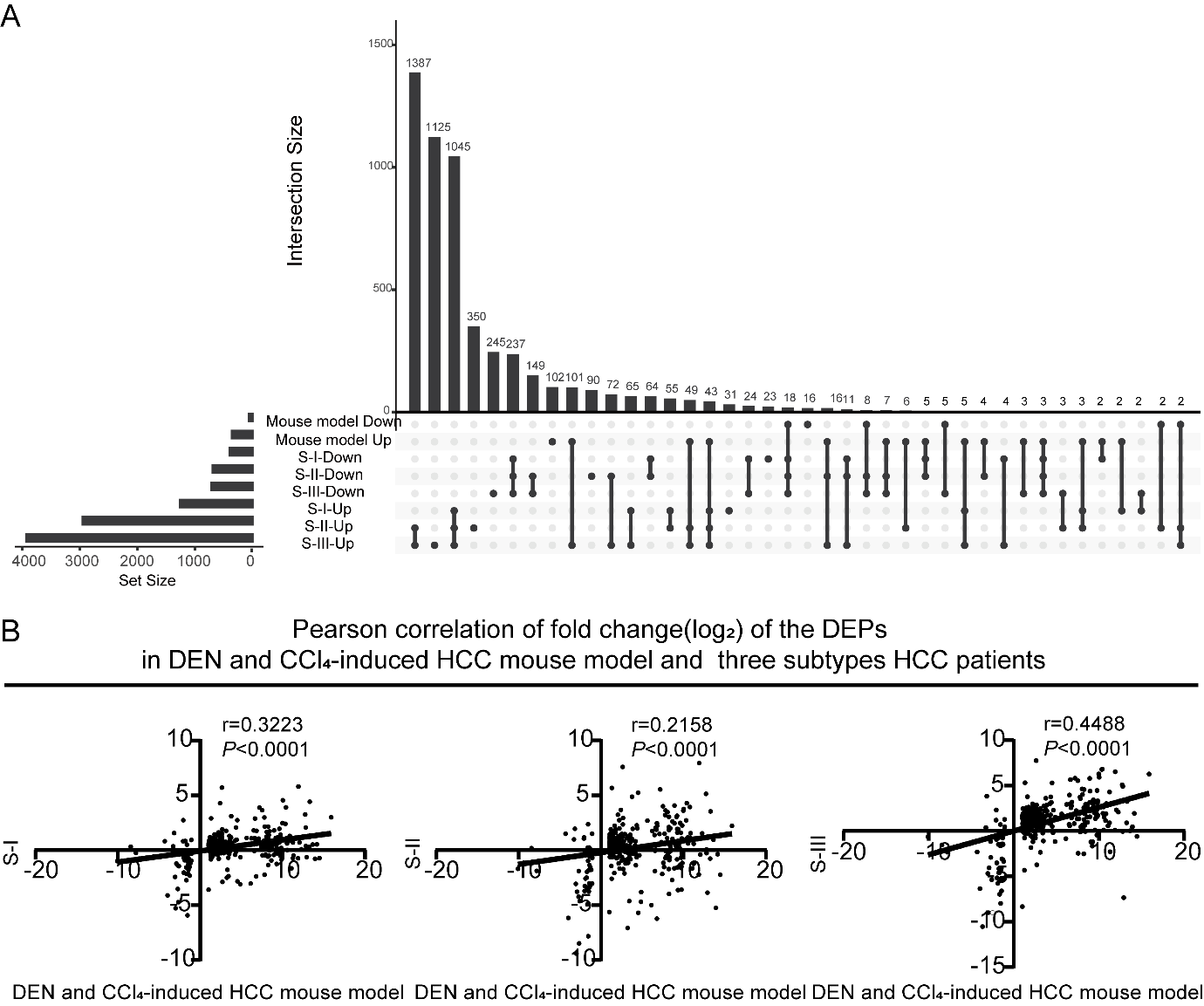


**Supplementary Figure 1. Correlation of differentially expressed proteins in DEN and CCl_4_-induced HCC mouse model and HCC clinical samples.** (a) Upset plots of significantly up-regulated and down-regulated proteins in tumor tissues of DEN and CCl_4_-induced HCC mouse model and significantly up-regulated and down-regulated proteins in tumor tissues of three subtypes of Jiang et al.’s HCC cohort. (b) Pearson correlation of fold change(log2) of the DEPs in DEN and CCl_4_-induced HCC mouse model and three subtypes of Jiang et al.’s HCC cohort.


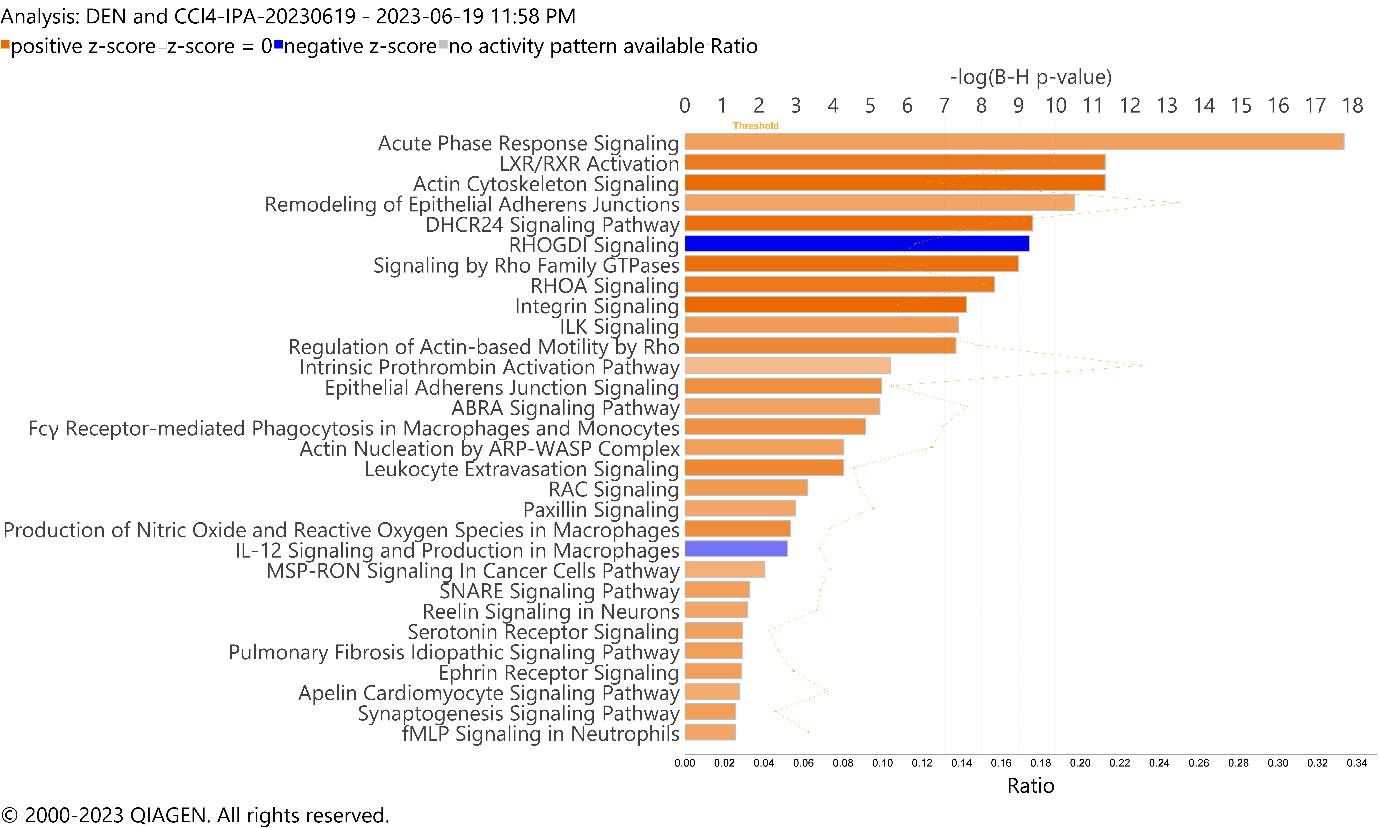


**Supplementary Figure 2. IPA analysis of proteomic data from DEN and CCl_4_-induced HCC mouse model.** Enrichment of Canonical Pathways. Blue bars represent negatively regulated pathways and orange bars represent positively regulated entries. -log (BHp) >1.3; Z score≥2.


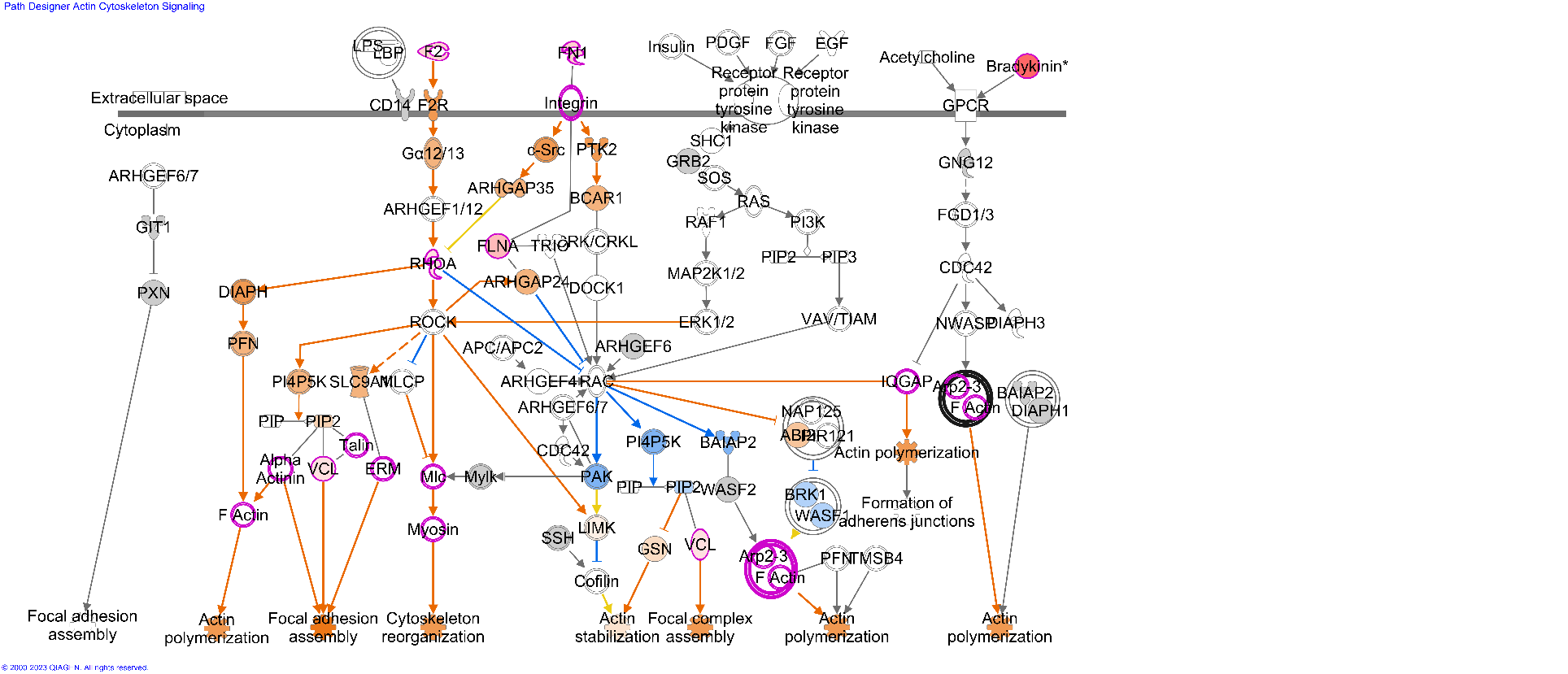


**Supplementary Figure 3. Actin Cytoskeleton Signaling expression and activation in DEN and CCl_4_-induced HCC mouse model tumor tissues.** Purple represents significant up-regulation, blue represents significant down-regulation, and orange represents positive activation.


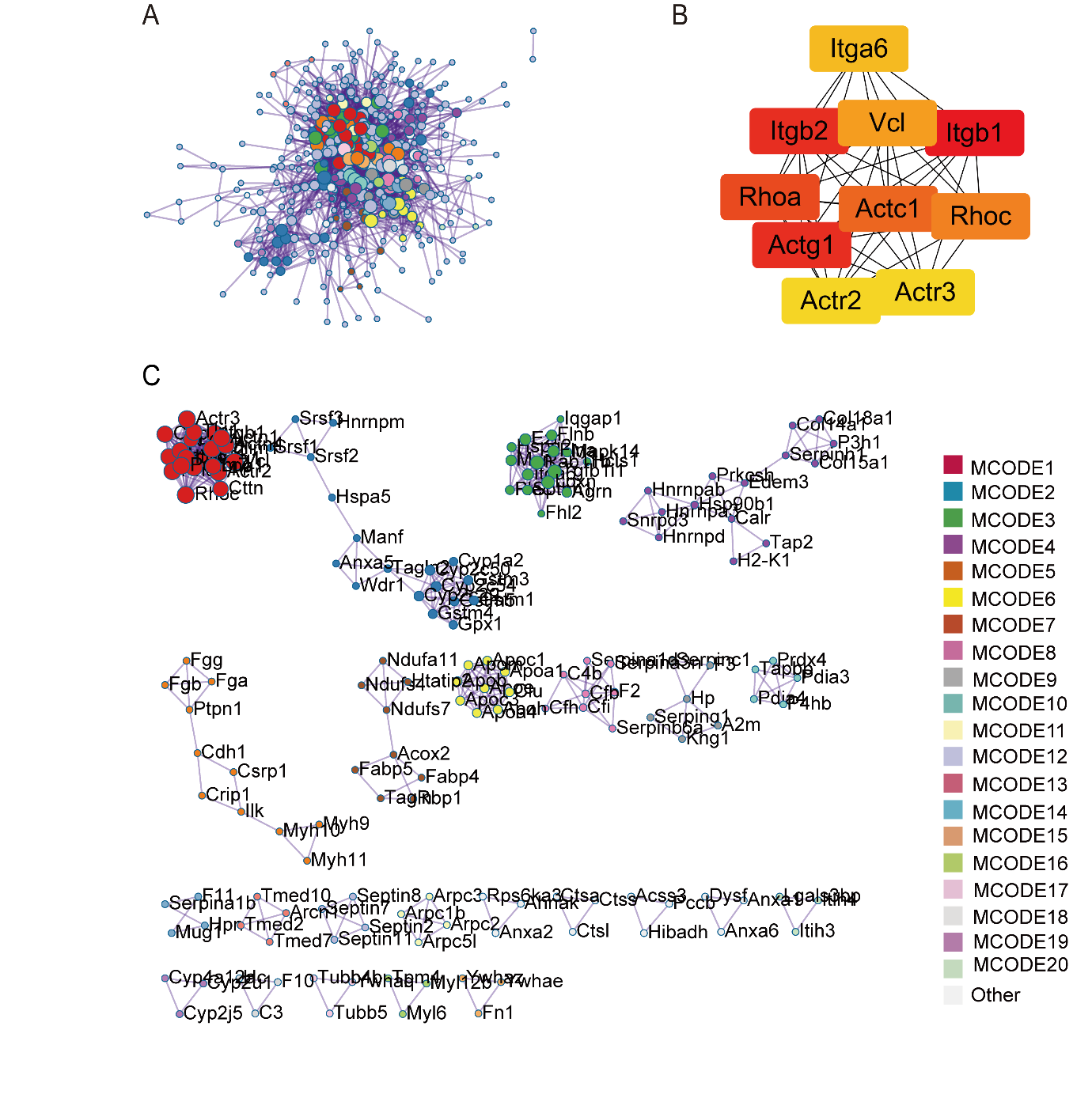


**Supplementary Figure 4. Protein-protein interaction network construction.** (a) PPI network of DEPs constructed by Metascape. (b) Top 10 proteins of PPI network ranked by Degree. (c) Metascape performed the MCODE algorithm on the DEPs and obtained 23 MCODEs.


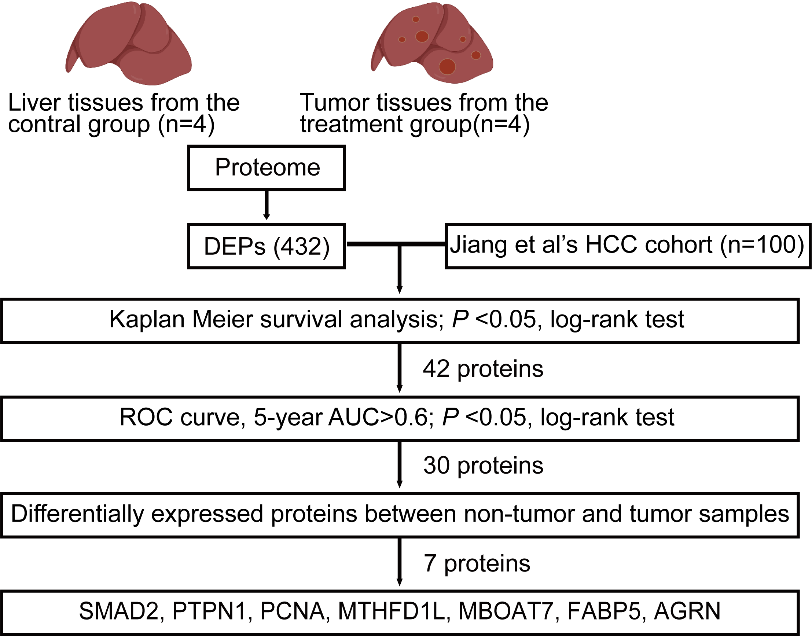


**Supplementary Figure 5. Screening for DEP proteins with significant prognostic value.** Schematic diagram of the process for screening proteins with prognostic value in DEPs in combination with Jiang et al.'s HCC cohort.


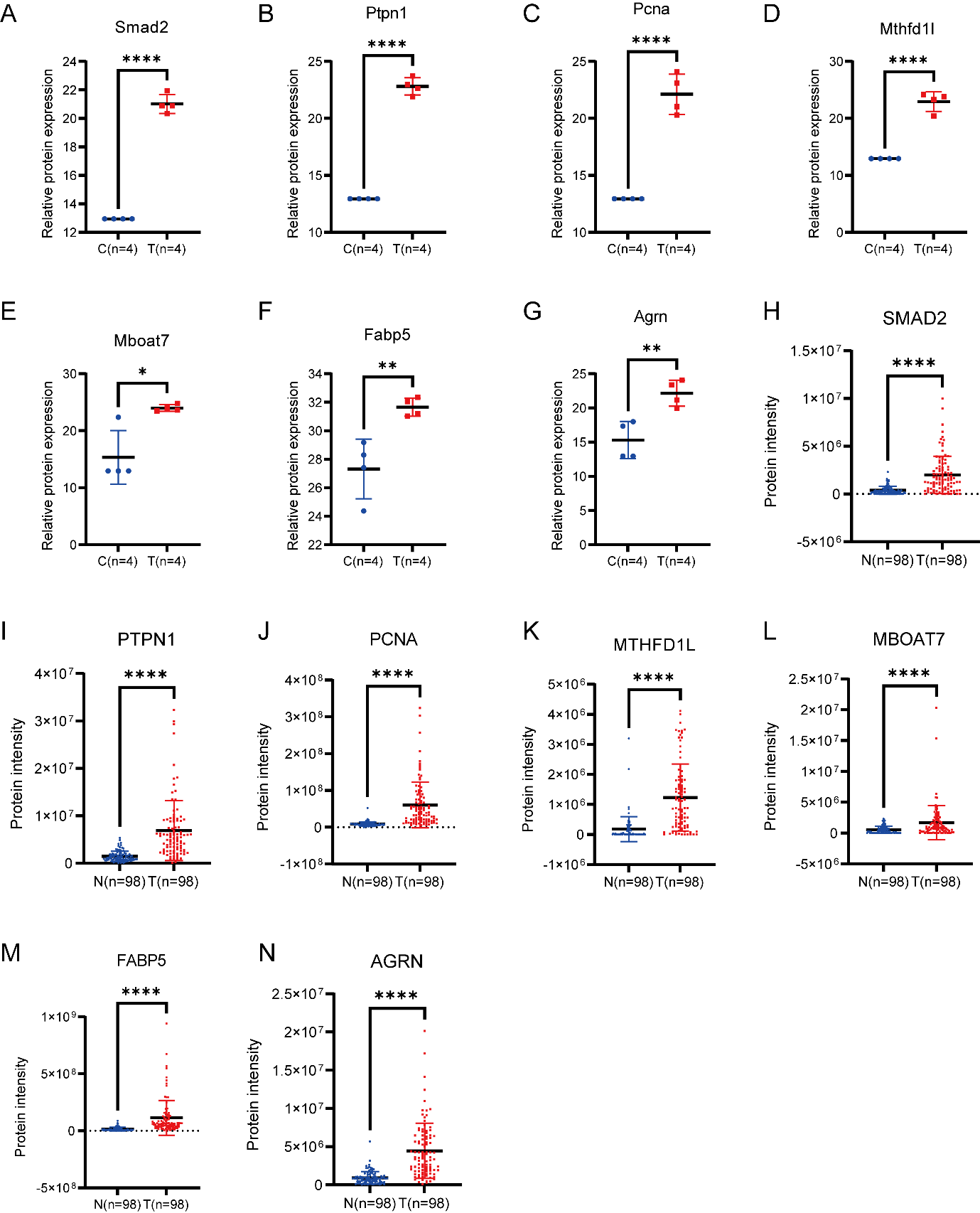


**Supplementary Figure 6. Expression of seven proteins with significant prognostic value in HCC.** (a)-(g) Expression of (a) MAD2, (b) PTPN1, (c) PCNA, (d) MTHFD1L, (e) MBOAT7, (f) FABP5, and (g) AGRN in liver tissue samples from control mice (C, blue) and tumor tissue samples from model mice (T, red). h-n Expression of (h) MAD2, (i) PTPN1, (j) PCNA, (k) MTHFD1L, (l) MBOAT7, (m) FABP5, and (n) AGRN in non-tumor tissue (N, blue) and tumor tissue (T, red) of HCC clinical samples from Jiang et al.'s HCC cohort. **P* < 0.05, ***P* < 0.01 and ****P* < 0.001.


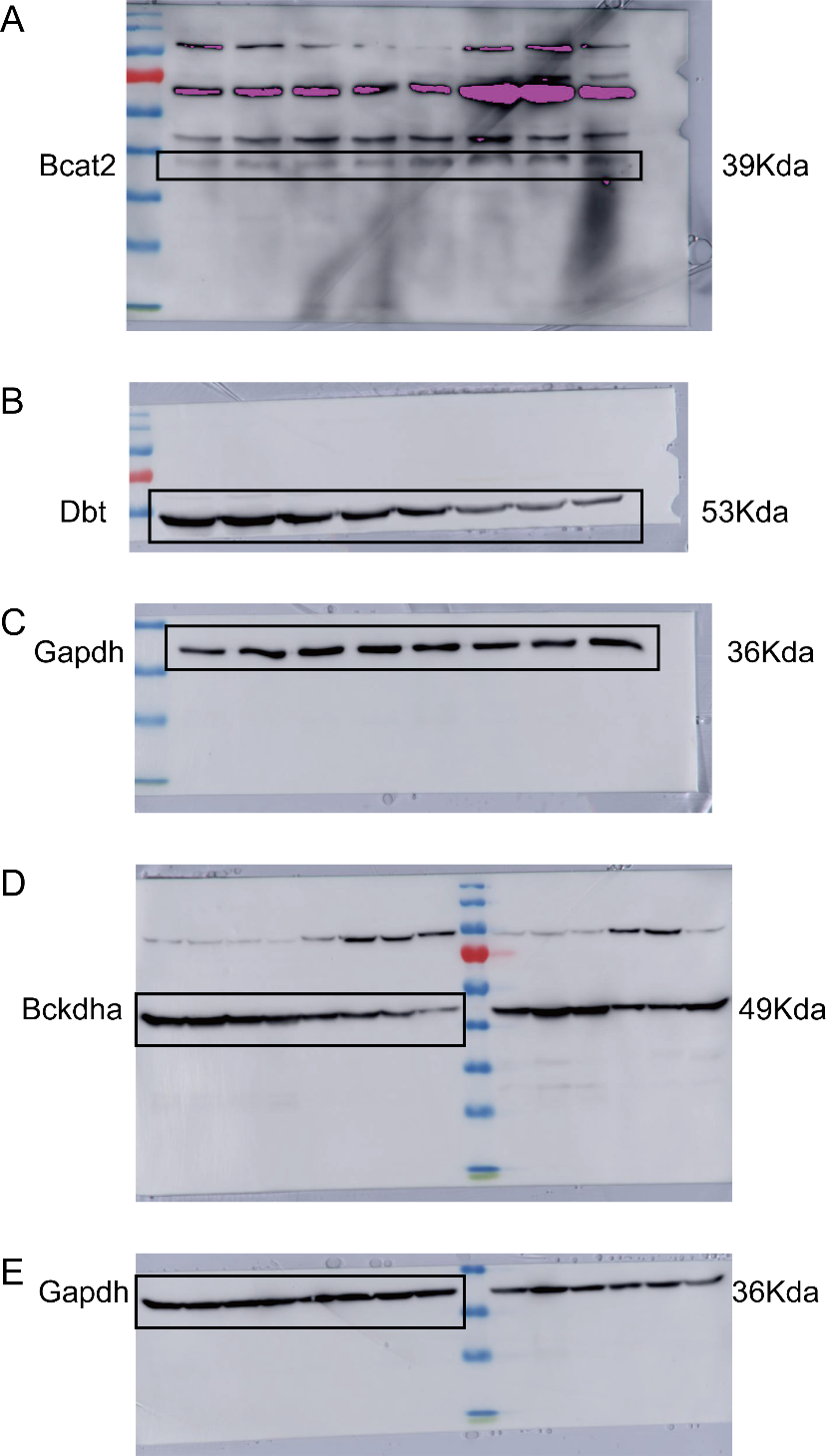


**Supplementary Figure 7. Representative full-****length immunoblots.** These black outlined boxes correspond to the cropped portion of the imprint shown in Fig.5A-B. (a) Representative full-length immunoblots of Bcat2. (b) and (c) show the results of (a) membrane after stripping the anti-Bcat2 antibody was cut into two parts and incubated with anti-Dbt antibody (b) and anti-Gapdh antibody (c), respectively. (d) and (e) are the same membrane cut into two parts and incubated with anti-Bckdha antibody (d) and anti-Gapdh (e) antibody, respectively. The samples were from the same experiment and the gels were processed in parallel.
